# A taxonomic revision of Tyrini of the Oriental region. V. Revision of the genus *Lasinus* Sharp, 1874 (Coleoptera, Staphylinidae, Pselaphinae)

**DOI:** 10.3897/zookeys.340.5980

**Published:** 2013-10-04

**Authors:** Rostislav Bekchiev, Peter Hlaváč, Shûhei Nomura

**Affiliations:** 1 National Museum of Natural History, 1 Tsar Osvoboditel Blvd, 1000 Sofia, Bulgaria; 2 Czech University of Life Sciences, Faculty of Forestry and Wood Sciences, Department of Forest Protection and Entomology, Kamýcká 1176, CZ-165 21 Praha 6-Suchdol, Czech Republic; 3 Department of Zoology, National Museum of Nature and Science, 4–1–1 Amakubo, Tsukuba, Ibaraki, 305–0005 Japan

**Keywords:** Tyrini, taxonomy, revision, new species, Russia - Kuril Islands, China, Vietnam, Japan

## Abstract

The genus *Lasinus* Sharp, 1874 of the *Pselaphodes* complex of genera (Pselaphitae: Tyrini: Tyrina) is revised. The three so far known species, *Lasinus mandarinus* Raffray, 1890, *Lasinus monticola* Sawada, 1961 and *Lasinus spinosus* Sharp, 1874 are redescribed. Eight new species, *Lasinus sinicus* **sp. n.** from China, *Lasinus mikado* **sp. n.**, *Lasinus yamamotoi* **sp. n.**, *Lasinus inexpectatus* **sp. n.**, *Lasinus yakushimanus* **sp. n.**, *Lasinus amamianus* **sp. n.**, *Lasinus saoriae* **sp. n.**, and *Lasinus okinawanus* **sp. n.** from Japan, are described. And all species are illustrated. Lectotypes are designated for *Lasinus mandarinus* and *Lasinus spinosus*. An identification key to species of the genus *Lasinus* is provided.

## Introduction

The genus *Lasinus* was erected by Sharp (1874) to accommodate his new species *Lasinus spinosus* from Japan. Another new species, *Lasinus mandarinus* was added by Raffray (1890) from the northern Vietnam. The last known species, *Lasinus monticola*,was described from Japan by Sawada (1961) who also provided illustrations of aedeagi of the presumed *Lasinus spinosus*. The genus was studied by the second author (Hlaváč 2003) and was included in the *Pselaphodes* complex of genera of the subtribe Tyrina, tribe Tyrini.

The purpose of this paper is the revision of the genus, the description of eight new species, as well as to provide a key for the identification of all species of the genus.

## Materials and methods

Dry-mounted specimens were relaxed in warm water. Dissections were made using standard techniques, genitalia and small parts were mounted in Euparal or Canada balsam on acetate labels which are pinned together with the specimens. Leica S8APO microscope was used for the study. All photos were done by microscope Olympus SZ 61 with camera Olympus Colorview I. The width of head is measured through eyes.

The material used in this study is deposited in the following public and private collections:

BMNH Natural History Museum, London, United Kingdom.

MCSN Museo Civico di Storia Naturale “G. Doria”, Genova, Italy.

MNHN Muséum National d’Histoire Naturelle, Paris, France.

MHNG Muséum d’histoire naturelle de la ville de Genève, Switzerland

NMNH National Museum of Natural History, Sofia, Bulgaria

NHMW Naturhistorisches Museum, Wien, Austria

NSMT National Museum of Nature and Science, Tsukuba, Japan.

PCPH Peter Hlaváč private collection, Prague, Czech Republic

PCSK Sergei Kurbatov private collection, Moscow, Russia.

Other abbreviations and symbols used in the text: p (printed); h (hand-written); / (used to separate different labels). All paratypes bear the following red label: PARATYPE *Genus species* sp. n., Bekchiev, Hlaváč & Nomura det., 2013.

## Taxonomy

### Genus Lasinus

Sharp

http://species-id.net/wiki/Lasinus

Lasinus Sharp, 1874: 106. Type species: *Lasinus spinosus* Sharp, 1874 (original designation, gender masculine).
Lasinus Sharp: Jeannel 1958: 121.
Lasinus Sharp: Hlaváč 2003: 286 (redescription).

#### Diagnosis.

The genus *Lasinus* can be readily separated from the other genera of the *Pselaphodes* complex (Hlaváč 2003) by a combination of the following characters: 1) head with well-defined setose frontal and vertexal foveae, 2) maxillary palpi small, with palpomeres III–IV symmetrical, neither roundly expanded nor projecting laterally, 3) antennal club three-segmented, with antennomeres VIII–IX often modified in males, 4) pronotal lateral and median foveae well-defined, 5) pronotum lacking antebasal sulcus connecting foveae, 6) pronotal longitudinal sulcus present, well- to weakly-defined, 7) tarsal segments II linear, segments III inserted at the apex of the II, 8) basal carinae on the first visible tergite (IV) present, short, 9) median setose metaventral fovea absent, 10) metaventral horny processes reduced to short, stout protuberances.

#### Redescription.

Length 2.5–3.8 mm. Head lacking dorsally visible postgenae, with well-defined setose frontal and vertexal foveae, maxillary palpi small, symmetric. Antennae with scapes distinctly longer than pedicels, club three-segmented, antennomeres VIII–IX often modified in males, species characteristic. Pronotum with well-defined median and lateral foveae, longitudinal sulcus sometimes weakly-defined but always present, antebasal transverse sulcus absent. Elytra with two basal foveae and two striae, lacking carinae, punctate, covered by short, golden pubescence. Metaventrite with two stout, short protuberances instead of long horn-like processes, metaventral apex sharp, with shallow excavation, surface punctate and pubescent, median metaventral fovea absent. Legs long and slender, roughly punctate and pubescent, protrochanters, mesotrochanters, profemora and mesofemora with spines of various shape and length, metatrochanters and metafemora lacking spines, tarsomeres II linear, tarsomeres III inserted at the apex of II.

#### Sexual dimorphism.

Females of all species bear on mesotrochanters one or two more spines than males, females antennomeres VII–IX simple, without modification.

#### Relationship.

Due to the simple second tarsal segments and the absence of the median metaventral foveae, *Lasinus* is most closely related to the genera *Paralasinus* Hlaváč & Nomura, 2001, *Pselaphodes* Westwood, 1870 and *Dayao* Yin, Li & Zhao, 2011. From the latter two genera *Lasinus* can be separated by the completely symmetric palpomeres II–IV. In *Pselaphodes* and *Dayao*, the maxillary palpi have at least some palpomeres II–IV asymmetric, roundly expanded, or slightly to distinctly projecting laterally. *Lasinus* differs from *Paralasinus* by the pronotum lacking an antebasal sulcus, which is present in *Paralasinus*.

#### Habitat.

Members of the genus are usually collected by sifting leaf-litter in forested areas.

**Distribution.** China, Vietnam, Japan, Russia (Kuril Islands).

#### Key to species of *Lasinus*

**Table:** 

1	Pronotum with prominent lateral swellings before lateral foveae (Figs 1, 4, 9). Body length 3.25–3.60 mm. Species from Vietnam or China	2
–	Pronotum evenly rounded (Figs 5, 8) or with weak swellings (Fig. 10) before lateral foveae. Body length 2.70–3.30 mm. Species from Japan	3
2	Antennomeres X elongate, about 1.6 times longer than wide (Fig. 1). (male unknown). Vietnam	*Lasinus mandarinus*
–	Antennomeres X short, about as long as wide in both sexes (Fig. 4). China	*Lasinus sinicus*
3	Antennomeres IX in male simple, only slightly obliquely trucate apically	4
–	Antennomeres IX in male modified, with well-defined sexual character	5
4	Antennomeres VII 1.3 times longer than wide; antennal club as in Fig. 15	*Lasinus inexpectatus*
–	Antennomeres VII 1.15 times longer than wide; antenal club as in Fig. 14	*Lasinus yamamotoi*
5	Antennomeres IX with well-developed preapical tubercule	6
–	Antennomeres IX with different type of ornamentation	7
6	Antennomeres VIII 1.16 times longer than wide; antennal club as in Fig. 17	*Lasinus amamianus*
–	Antennomeres VIII 0.9 times longer than wide; antennal club as in Fig. 18	*Lasinus saoriae*
7	Antennomeres IX with shallow concavity, lacking apical nail-shaped protuberance	8
–	Antennomeres IX with apical nail-shaped protuberance	9
8	Antennomeres IX almost rectangular; antennal club as in Fig. 11	*Lasinus spinosus*
–	Antennomeres IX with strong, internal obliquity at apex; antennal club as in Fig. 19	*Lasinus okinawanus*
9	Antennomeres IX with deep concavity; antennal club as in Fig. 13	*Lasinus mikado*
–	Antennomeres IX with shallow concavity; antennal club as in Figs 12–16	10
10	Genae angulate, with triangular, prominent protuberance (Fig. 6); pronotum evenly rounded before lateral fovea (Fig. 8); antennal club as in Fig. 12; aedeagus as in Fig. 21	*Lasinus monticola*
–	Genae convex, with weak protuberance (Fig. 7); pronotum with weak swellings before lateral fovea (Fig. 10); antenal club as in Fig. 16. Aedeagus as in Fig. 25	*Lasinus yakushimanus*

### Lasinus mandarinus

Raffray, 1890

http://species-id.net/wiki/Lasinus_mandarinus

Fig. 1

Lasinus mandarinus Raffray, 1890: 212, Pl. III, fig. 16.

#### Type locality.

Tonkin (Ha Noi, Vietnam).

#### Material examined

(4 ♀♀). LECTOTYPE, ♀, here designated: (h) Tonkin / red label (p) TYPE / (h) *Lasinus mandarinus* (p) A. Raffray det. / (p) MUSÉUM PARIS, 1917, COLL. A. RAFFRAY / red label (p) LECTOTYPE *Lasinus mandarinus* Raffray, Bekchiev, Hlaváč & Nomura des., 2013. (MNHN). PARALECTOTYPES, 3 ♀♀: same data as lectotype, bearing the following red label: (p) PARALECTOTYPE *Lasinus mandarinus* Raffray, Bekchiev, Hlaváč & Nomura des., 2013. (MNHN).

#### Lectotype designation.

Redescription of this species given below is based on four females deposited in MNHN having a status of syntypes. Raffray (1890) also mentioned four females in his original description. One female is here designated as lectotype, and three remaining females paralectotypes, in order to ensure the stability of nomenclature and provide a unique name-bearing type for *Lasinus mandarinus*.

#### Description.

**Figures 1–5.**
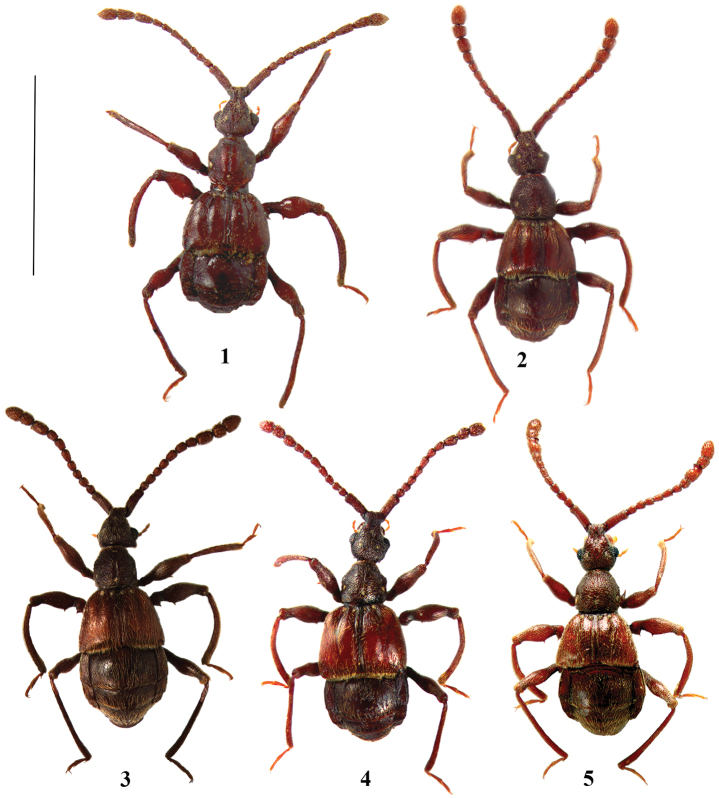
Habitus of *Lasinus* species. **1** *Lasinus mandarinus* **2** *Lasinus spinosus* **3** *Lasinus monticola* **4** *Lasinus sinicus* **5** *Lasinus mikado*. Scale – 3.0 mm.

Body (Fig. 1) unicoloured, dark brown, maxillary palpi yellow, length 3.30–3.60 mm.

Head elongate, about 1.15 times longer than wide, slightly longer than pronotum; median sulcus well-defined along whole length of head. Genae simple, without protuberance.

Antennae very long, about 2.3–2.4 mm; scapes about 5 times longer than pedicels, pedicels short, as long as wide; antennomeres III–VII subequal in length, slightly shorter than V; antennomeres VI–VIII about as long as pedicels; IX slender, 2.25 times longer than wide, 1.10 times as long as X; late about 1.6 times longer than wide; terminal segments 1.2 times as long as X and 1.6 times longer than wide.

Pronotum as long as wide, gibbose, with prominent lateral swellings before lateral foveae; lateral and median setose foveae well-defined; median sulcus originates in median fovea and almost reaching anterior margin of pronotum.

Legs long and slender; protrochanters with small apical spine; profemora with long spine before middle; mesotrochanters at apex with two spines, outer one fairly bigger; mesofemora with minuscule spine at basal third.

First visible abdominal tergite (IV) glabrous, slightly more than two times longer than second (V); short basal carinae well-defined, distance between carinae 0.4 of maximal tergal width.

#### Differential diagnosis.

*Lasinus mandarinus* is close to *Lasinus sinicus* by the pronotum with prominent lateral swellings before lateral foveae, but it differs from the latter by the proportion of antennomere X which is 1.6 times as long as wide.

#### Distribution.

Vietnam (Tonkin, Ha Noi).

### Lasinus spinosus

Sharp

http://species-id.net/wiki/Lasinus_spinosus

Figs 2 , 11 , 20

Lasinus spinosus Sharp, 1874: 106.
Lasinus spinosus Sharp: Waterhouse 1882, 90: pt. 21, pl. 146, fig. 3.

#### Type locality.

Nagasaki, Suwo-sama (=Suwa shrine).

#### Type material examined.

LECTOTYPE, ♂, here designated: (h) *Lasinus spinosus*. Type D.S. Japan. Lewis. [label where the type specimen was originaly mounted] / (h) *Lasinus spinosus* ♂ (p) TYPE (h) D. S. / (p) Japan. G. Lewis / round label with red margin (p) TYPE / round label with blue margin (p) SYNTYPE / (p) Sharp Coll. 1905-313. / red label (p) LECTOTYPE *Lasinus spinosus* Sharp, Bekchiev, Hlaváč & Nomura, des., 2013 (BMNH). PARALECTOTYPE, 1 ♀, here designated: (h) *Lasinus spinosus* ♀ (p) TYPE (h) D. S. / (p) Japan. G. Lewis / round label with blue margin (p) SYNTYPE / red label (p) PARALECTOTYPE *Lasinus spinosus* Sharp, Bekchiev, Hlaváč & Nomura, des., 2013 (BMNH).

#### Other material examined.

(8 ♂♂, 9 ♀♀). (1 ♂) Japan, Saga Pref., Kashima City, Mt. Kyogatake., 19.X.1986, S. Nomura leg.; (1 ♂) Japan, Fukuoka Pref., Hiko-san Mts., 3.V.1983, S. Nomura leg.; (1 ♂) Japan, Kumamoto Pref., Ueki-cho, 10.IV.1981, S. Naomi leg.; (1 ♂, 1 ♀) Japan, Kyushu, Nagasaki Pref., Isahaya-shi, Jôyama, Atagoyama, 18.III.1998, S. Nomura leg.; (1 ♂) Japan, Kyushu, Oita Pref., Shonai-machi, Nishi-Ohara, 20.VI.1998, K. Ôtsuka leg.; (1 ♂, 1 ♀) Japan, Kyushu, Miyazaki Pref., Tano-cho, Aoidake, 6.IX.1993, S. Nomura leg.; (1 ♂, 1 ♀) Japan, Fukuoka, Hikosan Mts., 27.XII.1982, S. Nomura leg.; (1 ♂, 1 ♀) Japan, Nagasaki City, Suwa Shrine, 2.V.1985, S. Nomura leg.; (1 ♀) Japan, Miyazaki Pref., Kiyotake-cho, Kaeda vall., 27.IV.1993, S. Nomura leg.; (2 ♀♀) Japan, Miyazaki Pref., Aya-Minami, 9.V.1985, S. Nomura leg.; (2 ♀♀) Japan, Kyushu, Oita Pref., Kujû Mts., Makinoto pass., 14.X.1991, S. Nomura leg. (NSMT, PCPH, NMNH, PCSK).

#### Lectotype designation.

Redescription of this species given below is based on one male and one female deposited in BMNH having a status of syntypes. Sharp (1874) mentioned three specimens in his original description. One male is here designated as lectotype, another female is paralectotype, in order to ensure the stability of nomenclature and provide a unique name-bearing type for *Lasinus spinosus*.

#### Description.

Body (Fig. 2) unicoloured, reddish-brown, maxillary palpi yellow, length 2.90–3.10 mm.

Head elongate, about 1.15 times longer than wide, slightly longer than pronotum; median sulcus visible on rostrum and on vertex reaching level of vertexal foveae. Genae with weak protuberance, covered with erected, dense golden setae.

Antennae about 2.02 mm long (Fig. 11); scapes long, about 3.7 times longer than pedicels; pedicels about 1.18 times shorter than antennomeres III; antennomeres IV and V as long as wide; antennomeres VI 1.22 times shorter than pedicels; antennomeres VII 1.25 times shorter than VI; antennomeres VIII about 1.27 times longer than and distinctly wider than VII; IX about 1.5 times longer than wide and about same length as terminal antennomeres, IX in male with small and shallow discoidal plate on apical half bearing small pore-like structure with one long seta, in female unmodified; antennomeres X quadrate, 1.5 times shorter than IX; terminal antennomeres 1.6 times longer than X and about 1.5 times longer than wide.

Pronotum about as wide as long, wrinkly, evenly rounded before lateral foveae; lateral and median setose foveae well-defined; median longitudinal sulcus very thin, but distinct.

Legs long and slender; protrochanters with large apical spine; profemora with long spine in middle of its length; mesotrochanters at apex with one small (male) or two (female) spines, in some cases one spine is slightly stronger; mesofemora with minuscule spine at basal third.

Abdomen slightly wider than elytra, first visible abdominal tergite (IV) finely punctate with dense and short golden setae, about 4 times as long as next tergite, basal carinae very short, distance between carinae 0.4 of maximal tergal width. Aedeagus (Fig. 20) 0.64 mm long; median lobe weakly narrowed apically, with short and large apical lobe; endophallus with two spines and one lamella; dorsal spine very big, enlarged at apex, forming large plate; ventral spine long, acute at apex; lamella small finely dentate in apical part; parameres short and slender, not overlapping apical lobe.

#### Differential diagnosis.

*Lasinus spinosus* shares with *Lasinus monticola*, *Lasinus mikado*, *Lasinus inexpectatus*, *Lasinus saoriae* and *Lasinus yamamotoi* the evenly rounded pronotal lateral margins, but differs from all of these species by the shape of the antennae and aedeagus.

#### Distribution.

Japan (Kyushu).

### Lasinus monticola

Sawada

http://species-id.net/wiki/Lasinus_monticola

**Figures 6–7.**
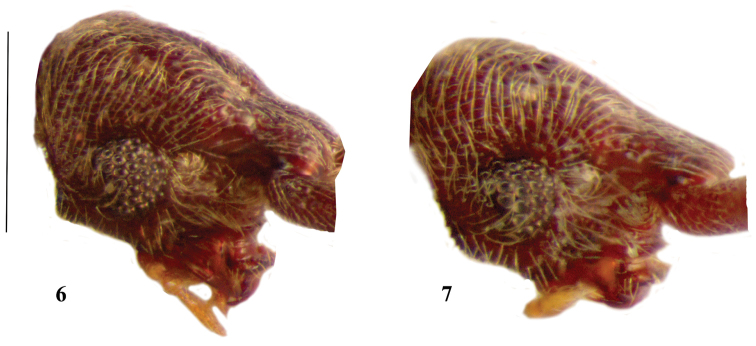
Head of *Lasinus monticola* (**6**) and *Lasinus yakushimanus* (**7**) lateral view. Scale – 0.4 mm.

**Figures 8–10.**
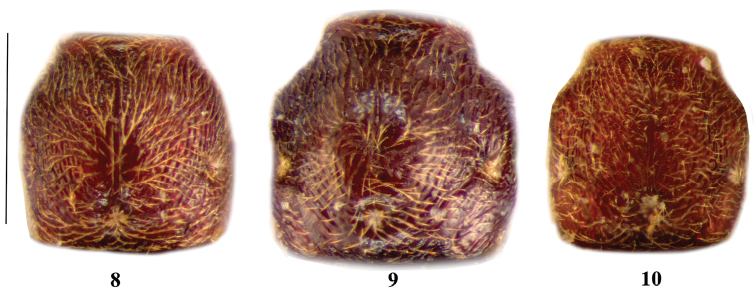
Pronotum of *Lasinus monticola* (**8**) *Lasinus sinicus* (**9**) and *Lasinus yakushimanus* (**10**) dorsal view. Scale – 0.6 mm.

Figs 3 , 6 , 8 , 12 , 21

Lasinus monticola Sawada, 1961: 41; pl. 7, figs 1, 3, 4.

#### Type locality.

Hiko (900 m), Fukuoka, Kyushu.

#### Material examined.

(21 ♂♂, 19 ♀♀). (3 ♂♂, 1♀): Japan, Shikoku, Ehime Pref., Oda-cho, Mt. Odamiyama, Buna st., 2.IX.1993, E. Yamamoto leg.; (1 ♂) Nara, Nara Park, 8.VIII.1980, I. Löbl leg.; (1 ♀) Japan: Honshu, Kanagawa Pref., Aikawa-chô Mt., Hasuge-san, 7.I.2006, T. Lackner leg.; (1 ♂) Japan, Fukushima pref, Okutadami, Alizu, Mt. Asakusadake, 22.VII.1987, S. Nomura leg.; (5 ♂♂) Japan, Shimane Pref., Kanagi-machi, Atoyama, 9.V.1991, T. Nakamura leg.; (2 ♂♂) Japan, Ehime Pref., Narukawa-keikoku 600–700 m, 1.II.1997, M. Sakai leg.; (1 ♂) Japan, Ehime Pref, Komi, Yanadani, 2.X.1994, M. Sakai leg.; (1 ♂, 7 ♀♀) Japan, Kyushu, Kagoshima Pref., Kirishima, Kurinodake Spa, 8.III.1999, H. Hoshina leg.; (1 ♂) Japan, Kyushu, Miyazaki Pref., Tsuno-chô, Mt. Osuzuyama, 700 m, 8.IX.1994, S. Nomura leg.; (2 ♂♂) Japan, Kyushu, Miyazaki Pref., Takachiho-chô, Onino-iwaya, 3.XII.1994, S. Nomura leg.; (1 ♂) Japan, Kyushu, Miyazaki Pref., Wanizukayama Mts., 6.IX.1993, S. Nomura leg.; (1 ♂, 1 ♀) Japan, Kyushu, Miyazaki Pref., Aya-chô, 10.II.1994, S. Nomura leg.; (1 ♂) Japan, Kyushu, Kagoshima Pref., Aira-chô, 30.I.1985, T. Tanabe leg.; (1 ♀) Japan, Honshu, Tokyo Pref., Fussa-shi, Tamagawa Riverside, Mutsumi-bashi, 12.II.2007, S. Nomura leg.; (1 ♀) Japan, Honshu, Tokyo Pref., Okutama, Nippara, Ogawadani, 4.IV.2006, S. Nomura leg.; (4 ♀♀) Japan, Kyushu, Nagasaki Pref., Unzen Mt. Kinugasayama, 16.III.2007, S. Nomura leg.; (1 ♀) Japan, Kyushu, Kagoshima Pref., Osumi Mt., Hoyoshidake, 19.III.1994, S. Nomura leg.; (1 ♂) Japan, Kioto, 11.VI.1881, G. Lewis leg.; (2 ♀♀) Japan, Miyanoshita, 11.VI.1881, G. Lewis leg. (BMNH, NSMT, PCPH, NMNH, PCSK).

#### Description.

Body bicoloured (Fig. 3), darker brown with more reddish elytra, maxillary palpi yellow, length 2.80–3.30 mm.

Head elongate, about 1.06 times longer than wide and as long as pronotum; median sulcus weakly defined along whole length of head. Genae with triangular, prominent protuberance, covered with erect, dense golden setae (Fig. 6).

Antennae about 2.23 mm long (Fig. 12); scapes long, about 4 times longer than pedicels; pedicels shortest, quadrate and as long as IV and V each; antennomeres III about 1.25 times longer than pedicels; antennomeres VI–VII subequal in length; VIII slightly shorter than VII; IX about 1.5 times longer than wide, with apical, nail-shaped protuberance on ventral side in male, in female unmodified; antennomeres X quadrate, 1.25 times shorter than IX; terminal antennomeres 1.5 times longer than X and about 1.5 times longer than wide.

Pronotum slightly longer than wide, wrinkly, evenly rounded before lateral foveae (Fig. 8); lateral and median setose foveae well-defined; median sulcus weakly-defined.

**Figures 11–19.**
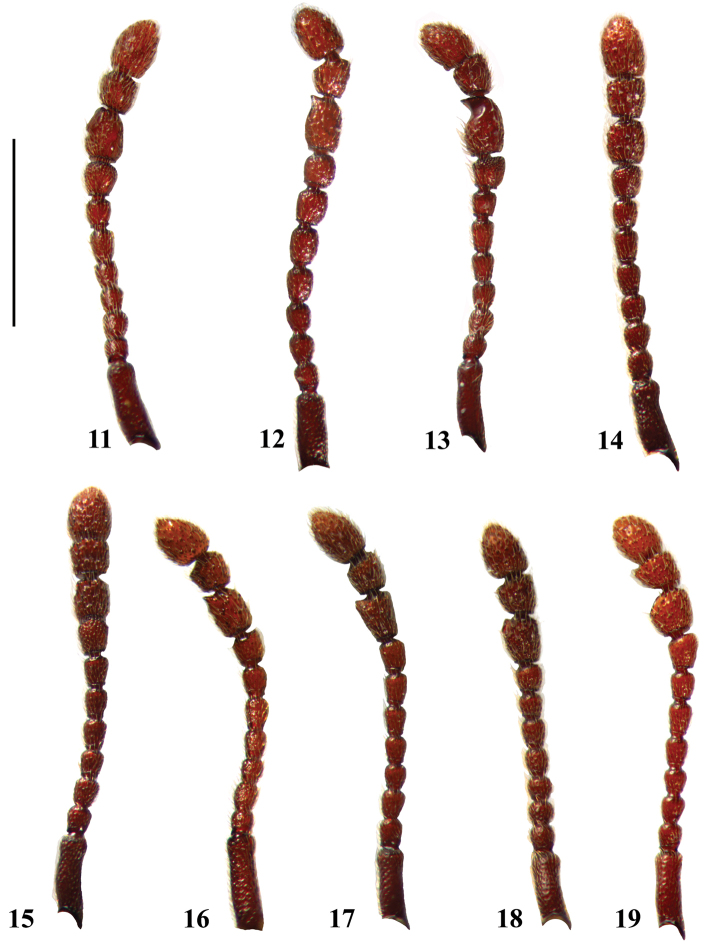
Antenae of *Lasinus* species (Japan). **11** *Lasinus spinosus* **12** *Lasinus monticola* **13** *Lasinus mikado* **14** *Lasinus yamamotoi* **15** *Lasinus inexpectatus* **16** *Lasinus yakushimanus* **17** *Lasinus ammamnianus* **18** *Lasinus saoriae* **19** *Lasinus okinawanus*. Scale – 1 mm.

Legs long and slender; protrochanters with small apical spine; profemora with long spine before middle; mesotrochanters at apex with two (males) minuscule spines or three (females) spines, median one minuscule; mesofemora with minuscule spine at basal third.

Abdomen slightly wider than elytra, first visible tergite (IV) about 3 times as long as second (V), finely punctate with dense, very short golden setae; basal carinae well-defined but very short, distance between carinae 0.5 of the maximal tergal width. Aedeagus (Fig. 21) 0.66 mm long; median lobe weakly narrowed apically, with short and large apical lobe, curved downwards in middle; endophallus with two spines and one large lamella; dorsal spine large, enlarged in middle to form broad plate, acutely angled at apex, with one small tooth in middle; ventral spine short, acute at apex; lamella large; parameres long, overlapping apical lobe, enlarged apically.

**Figures 20–23.**
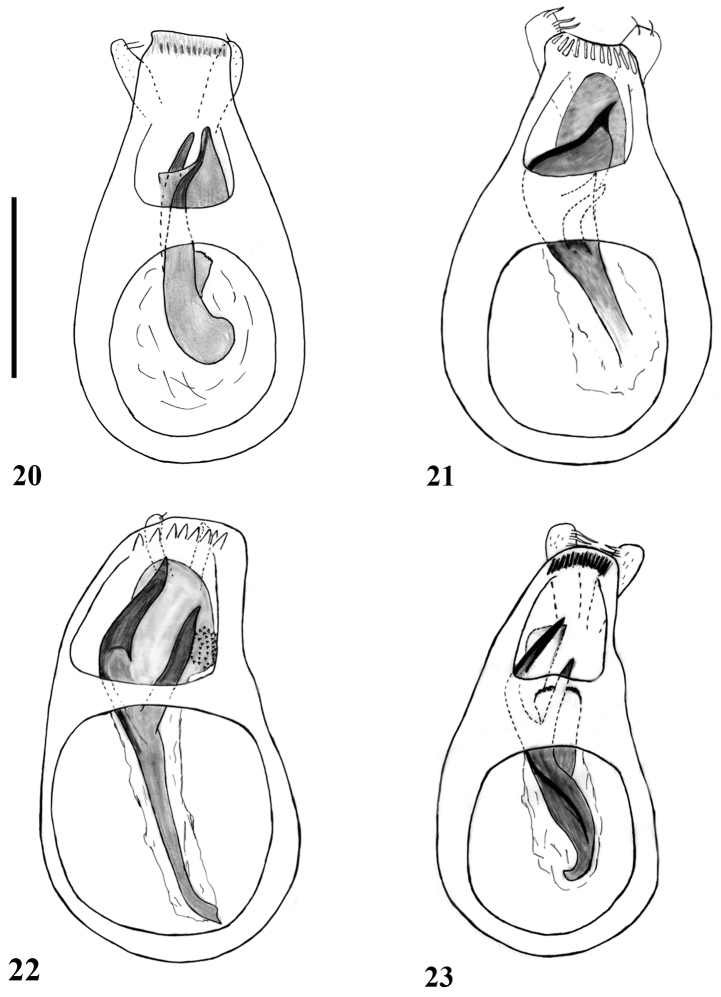
Aedeagi of *Lasinus* species (dorsal view). **20** *Lasinus spinosus* **21** *Lasinus monticola* **22** *Lasinus sinicus* **23** *Lasinus mikado*. Scale – 0.3 mm.

#### Differential diagnosis.

*Lasinus monticola* is close to *Lasinus mikado* and *Lasinus yakushimanus* by the presence of nail-shaped protuberance on the antennomeres IX,butit differs from *Lasinus yakushimanus* by the shape of the genal region of the head and the pronotum, and it differs from *Lasinus mikado* by the absence of a deep concavity on antennomeres IX. *Lasinus monticola* can be readily separated from both species also by the shape of aedeagus.

#### Distribution.

Japan (Honshu, Shikoku, Kyushu).

### Lasinus sinicus sp. n.

http://zoobank.org/2ADC741B-FF05-41DD-BE15-56412786449F

http://species-id.net/wiki/Lasinus_sinicus

Figs 4 , 9 , 22

#### Type material

(5 ♂♂, 3 ♀♀). HOLOTYPE, ♂, labelled as follows: (p) [Guanxi,CHINA], “locality in Chinese characters”, 25.V.1996, S. Uéno leg. / (p) ab. Liangshui 1710 m, Mt. Miao’ershan, Xing’an Xian. / red label (p) HOLOTYPE *Lasinus sinicus* sp. n., Bekchiev, Hlaváč & Nomura det., 2013. (NSMT). PARATYPES: 1♂: China, Vill, 86, Gansu: Mrijishan (h), 1000 m, Rougemont: 1 ♂, 2 ♀♀: same data as holotype, but specimens collected on 26.V.1996; 1 ♂, 1 ♀: China, Shaanxi, Nanwutaishan, 4.04.03, leg. Rougemont; 1 ♂: China, W Hubei, 21.VI–13.VII, Guanmenshan-1500 m, pit fall traps, 31.45N, 110.4E, leg. Jaroslav Turna, 2003. All paratypes bear the following red label: (p) PARATYPE *Lasinus sinicus* sp. n. Bekchiev, Hlaváč & Nomura det., 2013. (NSMT, MCSN, PCPH).

**Description.** Body (Fig. 4) bicolored, head, pronotum and abdomen almost black, elytra reddish-brown, maxillary palpi yellow, length 3.25–3.50 mm.

Head elongate, about 1.15 times longer than wide, slightly longer than pronotum; median sulcus absent along whole length of head. Genae simple, lacking protuberances.

Antennae long about 2.10 mm; scapes long, about 4 times longer than pedicels; pedicels shortest, 1.25 times shorter than antennomeres III; antennomeres III, IV, VII and VIII subequal in length, slightly shorter than V and VI; antennomeres XI 0.71 times longer than wide only slightly enlarged on the apex of antennomeres in male, in female unmodified; X 0.85 times longer than wide; XI 0.66 times longer than wide.

Pronotum about as long as wide, wrinkly, gibbose, with prominent lateral swellings before lateral foveae (Fig. 9); lateral and median setose foveae well-defined; median sulcus present only on disc, not originates from median fovea, very short and fine, largely separated from anterior margin of pronotum.

Legs long and slender; protrochanters with small apical spine; profemora with small spine in middle; mesotrochanters at apex with small median spine (male) or two (female) spines; mesofemora with small spine at basal third.

First visible abdominal tergite (IV) glabrous, very long, about 3.50 times longer than second (V); basal carinae very short, distance between carinae about 0.5 of maximal tergal width. Aedeagus (Fig. 22) 0.66 mm long; median lobe weakly narrowed apically, with short and very large apical lobe; endophallus with one large, bifid spine and one lamella; lamella large, with dentation on inner left part; parameres short and slender, not overlapping apical lobe.

#### Differential diagnosis.

*Lasinus sinicus* is close to *Lasinus mandarinus* by the similar shape of the pronotum with prominent lateral swellings before the lateral foveae. They can be separated from it by the proportion of antennomere X which is almost as long as wide in *Lasinus sinicus*.

#### Etymology.

The specific name is derived from China, where the species was discovered.

#### Distribution.

China (Guangxi, Gansu, Shaanxi, Hubei)

### Lasinus mikado sp. n.

http://zoobank.org/391BFF8A-01EF-4AE1-8AFF-22A9BDD369B3

http://species-id.net/wiki/Lasinus_mikado

Figs 5 , 13 , 23

Lasinus spinosus Sharp: Jeannel 1958: 121–122; figs 146, 147, 148.
Lasinus spinosus Sharp: Sawada 1961: 41; pl. 7, figs 5, 6.

#### Type material

(14 ♂♂, 17 ♀♀). HOLOTYPE, ♂, labelled as follows: (p) [Japan, Miyanoshita, Lewis], red label (p) HOLOTYPE *Lasinus mikado* sp. n., Bekchiev, Hlaváč & Nomura det., 2013. (BMNH); PARATYPES (2 ♂♂, 1♀): Japan, Nanatsukahara, Shobara City, Hiroshima Pref., 10.X.1987, I. Okamoto leg.; (1 ♂) Japan, Honshu, Chiyoda-ku, Tokyo Pref., Fukiage Gyoen, Imperial palace, 19.I.2001, S. Nomura leg.; (1 ♂) same data, 8.V.2001; (1 ♀) 18.XII.2003; (2 ♀♀) 2.II.2004; (3 ♀♀) 12.II.2007; (1 ♂) Japan, Honshu, Fussa-shi, Tokyo Pref., Tamagawa riverside, Mutsumi-bashi, 12.II.2007, S. Nomura leg.; (1 ♂) Japan, Honshu, Akiu-machi, Miyagi Pref., Futakuchi Valley, 27.VII.1990, S. Nomura leg.; (3 ♂♂, 1 ♀) Japan, Honshu, Saitama Pref., Ranzan-machi, Kagamata, 5.IV.1996, K. Toyoda leg.; (1 ♂) Japan, Honshu, Chiba Pref., Kôzaki-jinja, Kôzaki-machi, 14.X.2001, S. Nomura leg.; (1 ♂, 2♀♀) Japan, Honshu, Niiharu-mura, Gunma Pref., Hôshi-onsen, 600 m, 20.X.2001, S. Nomura leg.; (2 ♀♀) Japan, Niigata Pref., Kuroiwa, Shibata, 22.XI.1990, H. Koike leg.; (1 ♂) Japan, Nara, 27–31.VII.1980, C. Besuchet leg.; (1 ♂) Japan, Shikoku, Ishizuchi Mts., Omogo Valley, 700 m, 18-25.VIII.1980, J. Peck leg.; (1 ♂) Japan, Ôhira, Shimamaki-mura, Hokkaido, 4-18. VI. 1994, S. Hori leg.; (1 ♂) Japan, Teshio-gawa, Teshio-chô, Hokkaido, 22. VII. 1992, S. Hori leg.; (4 ♂♂, 2 ♀♀) Russia, Kunashir Island, Tretiakovo VIII., 20.VII.1990, S. Kurbatov leg.; (1 ♀) same data, 18.VII.1990; (1 ♂) Japan, 1890, Schönfeldt leg.; (7 ♂♂, 10 ♀♀) Japan, Kanagawa, Sauter leg., (BMNH, NSMT, NHMW, NMNH, MHNG, PCPH, PCSK).

#### Description.

Body (Fig. 5) unicoloured, reddish-brown, maxillary palpi light brown, length 2.87–3.1 mm.

Head elongate, about 1.10 times longer than wide, slightly longer than pronotum; median sulcus visible on rostrum, on vertex reaching level of vertexal foveae. Genae with weak protuberance, covered with erected, dense golden setae.

Antennae about 2.15 mm long (Fig. 13); scapes long, about 3.4 times longer than pedicels; pedicels about 1.18 times shorter than antennomeres III, antennomeres V and VI as long as pedicels; VII 1.25 times shorter than VI; antennomeres VIII about 1.27 times longer and distinctly wider than VII; antennomeres IX about 1.5 times longer than wide, about the same length as terminal antennomeres, in male with deep ventral concavity on apical half terminating with nail-shaped protuberance, in female unmodified; antennomere X quadrate, 1.5 times shorter than IX; terminal antennomeres 1.6 times as long as X and about 1.5 times longer than wide.

Pronotum about as wide as long, wrinkly, evenly rounded before lateral foveae; lateral and median setose foveae well-defined; median sulcus thin.

Legs long and slender; protrochanters with large apical spine; profemora with long spine in middle of its length; mesotrochanters at apex with two minuscule (male) or two strong (female) and one minuscule spine; mesofemora with minuscule spine at basal third.

Abdomen slightly wider than elytra; first visible abdominal tergite (IV) finely punctate, with dense and long golden setae, about 4 times longer than second visible tergite (V); basal carinae very short, distance between carinae 0.53 of maximal tergal width. Aedeagus (Fig. 23) 0.61 mm long; median lobe weakly narrowed apically, with long and relatively narrow apical lobe; endophallus with one bifid spine and two small lamellas; lamellas finely dentate on the apical part; parameres long, overlapping apical lobe, enlarged at apex.

#### Differential diagnosis.

*Lasinus mikado* is close to *Lasinus monticola* and *Lasinus yakushimanus* by the presence of a nail-shaped protuberance on antennomeres IX, it differs from both by the presence of a deep concavity on antennomeres IX and by the shape of aedeagus.

#### Etymology.

The name is derived from the Japanese word – „mikado“, meaning the Emperor of Japan.

#### Distribution.

Japan (Hokkaido, Honshu, Shikoku), Russia (Kuril Islands).

#### Remarks.

The original type series of *Lasinus spinosus* in the Sharp collection is in fact a mix of *Lasinus spinosus* and *Lasinus mikado* sp. n.

### Lasinus yamamotoi sp. n.

http://zoobank.org/79AC04BB-D1ED-4108-9984-DE09D9FC6B2D

http://species-id.net/wiki/Lasinus_yamamotoi

Figs 14 , 24

#### Type material.

(4 ♂♂, 1♀).HOLOTYPE, ♂, labelled as follows: (p) [Japan, Ehime, Nomura Dam, Nomura-cho, 27.V.1994, M. Sakai leg.] red label (p) HOLOTYPE *Lasinus yamamotoi* sp. n., Bekchiev, Hlaváč & Nomura det., 2013. (NSMT). PARATYPES: (2 ♂♂, 1♀); (1 ♂) same data with holotype; (1 ♀) same data with holotype but in 23.VII.1994; (1 ♂) Japan, Shikoku, Ehime Pref., Uchiko-chô, Shiromawari, 9.VII.1995, E. Yamamoto leg. (NSMT, PCPH, NMNH).

#### Description.

Body unicoloured, head, pronotum and abdomen reddish-brown, elytra slightly lighter, maxillary palpi yellow, length 2.85–2.95 mm.

Head elongate, 1.08 times longer than wide and as long as pronotum; median sulcus visible on rostrum, relatively shallow at level of vertexal foveae. Genae with weak protuberance, covered with erected, dense golden setae.

Antennae (Fig. 14) about 2.02 mm long; scapes long, about 2.85 times longer than pedicels; pedicels short, 1.42 times shorter than each of antennomeres III–IV; antennomeres V and VI of same length; antennomeres VII 1.22 times longer than wide; VIII longer than wide; XI longer than wide, simple, only slightly oblique on ventral side in male, in female unmodified; X slightly longer than wide; XI longer than wide.

Pronotum slightly longer than wide, surface evenly wrinkly, evenly rounded before lateral foveae; lateral foveae well-defined; median setose fovea small; median sulcus very thin.

Legs long and slender; protrochanters with long apical spine; profemora with short, strong spine in middle; mesotrochanters with one small (male) or two (female) spines; mesofemora with small spine at basal third.

**Figures 24–29.**
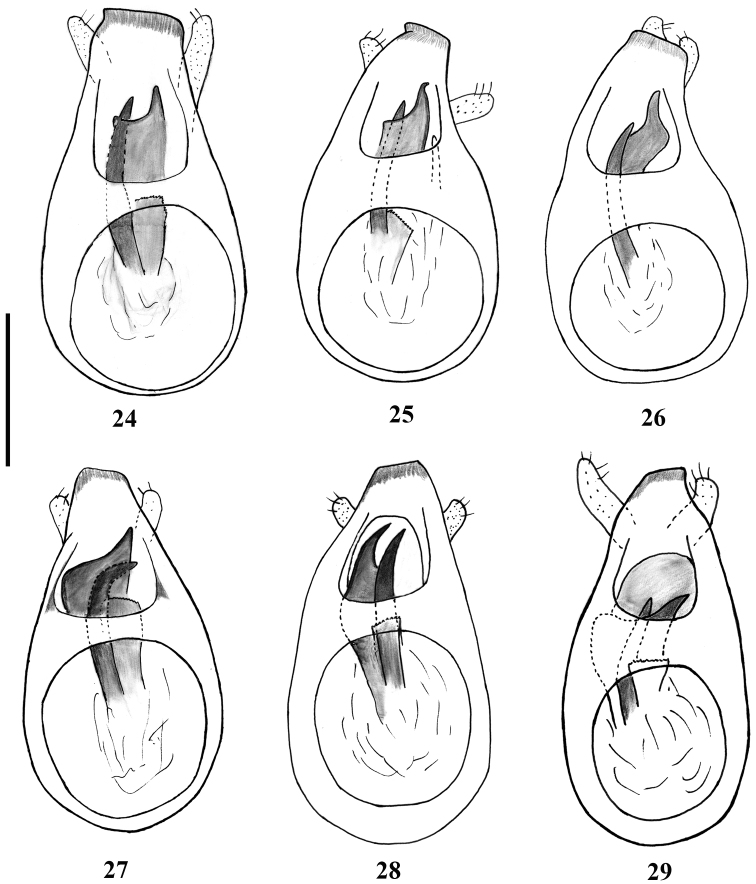
Aedeagi of *Lasinus* species (dorsal view). **24** *Lasinus yamamotoi* **25** *Lasinus inexpectatus* **26** *Lasinus yakushimanus* **27** *Lasinus amamianus* **28** *Lasinus saoriae* **29** *Lasinus okinawanus*. Scale – 0.3 mm.

First visible abdominal tergite (IV) very long, about four times longer than second visible tergite (V), with fine punctation in anterior part, disc glabrous, surrounded with short golden pubescence on sides; carinae short, distance between carinae 0.4 of maximal tergal width. Aedeagus (Fig. 24) 0.57 mm long; median lobe weakly narrowed apically, with large and relatively short apical lobe; endophallus with two spines and small lamella; ventral spine very big, forming large plate; dorsal spine big, curved, acute at apex; lamella small, finely dentate in apical part; parameres short and slender, reaching apical lobe, enlarged at apex.

#### Differential diagnosis.

*Lasinus yamamotoi* resembles *Lasinus inexpectatus* due to the unmodified antennal clubs in both sexes, but it can be distinguished from the latter by the proportion of the antennomeres VII and the shape of the aedeagus.

#### Etymology.

Patronimic, dedicated to Mr. Eiji Yamamoto (Japan).

#### Distribution.

Japan (Shikoku).

### Lasinus inexpectatus sp. n.

http://zoobank.org/DD3C5D54-9348-42C5-942B-E6BAC99ACA95

http://species-id.net/wiki/Lasinus_inexpectatus

Figs 15 , 25

#### Type material

(3 ♂♂, 1♀). HOLOTYPE, ♂, labelled as follows: (p) Japan, Kyushu, Miyazaki Pref., Aya-chô, Ôtsuribashi, 25.IV.1993, leg. S. Nomura. red label (p) HOLOTYPE, Lasinus inexpectatus sp. n., Bekchiev, Hlaváč & Nomura det. 2013 (NSMT). PARATYPES, (2 ♂♂, 1♀): 1 ♂, 1 ♀, same data as holotype, 1 ♂: (p) Japan, Kyushu, Miyazaki Pref., Nangoh-choh, Ohshima (3), leg. Atsushi Nagai (NSMT, NMNH, PCPH).

#### Description.

Body unicoloured, reddish-brown, elytra slightly lighter, maxillary palpi light brown, length 2.80–3.20 mm.

Head about 1.05 longer than wide, and as long as pronotum; median sulcus very weakly-defined, slightly visible only on rostrum, absent on vertex. Genae with weak protuberance, covered with erected, dense golden setae.

Antennae 2.20–2.24 mm long (Fig. 15); scapes long, about 3.25 times longer than pedicels; pedicels slightly shorter than antennomeres III–VII combined, which are subequal; VIII slightly longer and about 1.30 times wider than VII; antennomeres IX rectangular, simple, in male slightly shorter (0.18 mm) than in the female (0.24 mm); X about as wide as long; terminal antennomeres 1.60 times longer than X.

Pronotum 1.05 times longer than wide, wrinkly, evenly rounded before lateral foveae; lateral and median setose foveae well-defined; median sulcus thin, present only on disc.

Legs long and slender; protrochanters with large apical spine; profemora with long spine in middle of its length; mesotrochanters at apex with one minuscule (male) or two strong (female) spines; mesofemora with minuscule spine at basal third.

Abdomen slightly wider than elytra; first visible abdominal tergite (IV) finely punctate with dense and long golden setae on sides, disc glabrous, about 3 times longer than second visible tergite (V); basal carinae very short, almost invisible in males. Aedeagus (Fig. 25) 0.63 mm long; median lobe weakly narrowed apically, with long and narrow apical lobe; endophallus with three spines and small lamella; ventral spine large, forming large plate, acute and curved at apex; dorsal spine large, curved, acute at apex; lateral spine very slender and small, acute at the apex; lamella small, finely dentate on apical part; parameres short, unequal in length, not overlapping apical lobe, enlarged at apex.

#### Differential diagnosis.

*Lasinus inexpectatus* is close to *Lasinus sinicus* due to the simple antennomeres IX lacking any sexual character. The two species can be separated by the absence of swellings before lateral foveae on the pronotum in *Lasinus inexpectatus*. From all other Japanese species, *Lasinus inexpectatus* also differs by the shapes of the aedeagus and the antennae.

#### Etymology.

The name is realated with the „unexpected” discovery of this species.

#### Distribution.

Japan (Kyushu).

### Lasinus yakushimanus sp. n.

http://zoobank.org/2845C2F2-7942-499E-8D3E-7D5948F31728

http://species-id.net/wiki/Lasinus_yakushimanus

Figs 7 , 10 , 16 , 26

#### Type material.

(8 ♂♂, 7 ♀♀). HOLOTYPE, ♂, labelled as follows: (p) [Japan, Kyushu, Kagoshima Pref., Yakushima, Hananoegô, 17.III.2001, H. Hoshina leg.] red label (p) HOLOTYPE *Lasinus yakushimanus* sp. n., Bekchiev, Hlaváč & Nomura det., 2013 (NSMT). PARATYPES: (6 ♂♂, 3 ♀♀) same data as holotype; (1 ♂, 3♀♀) Japan, Kagoshima Pref., Yakushima, Mt. Nonkidake, 1350 m, 4.IX.2006, S. Nomura leg.; (1♀) Japan, Kagoshima Pref., Yakushima, Mt. Aikodake, 200 m, 5.IX.2006, S. Nomura leg. (NSMT, PCPH, NMNH, PCSK).

#### Description.

Body unicoloured, reddish-brown, maxillary palpi yellow dark, length 2.84–2.96 mm.

Head elongate, about 1.07–1.11 times longer than wide and about as long as pronotum; median sulcus visible on rostrum and on vertex reaching level of vertexal foveae. Genae with weak protuberance, covered with erected, dense golden setae (Fig. 7).

Antennae about 1.94 mm long (Fig. 16); scapes long, about 3.0 times longer than pedicels; pedicels as long as antennomeres III; antennomeres IV and V slightly longer than wide; antennomeres VI 1.62 times longer than wide; antennomeres VII as long as wide; antennomeres VIII as long as and slightly wider than VII; IX about 1.20 times longer than wide, in male with shallow ventral excavation on apical half terminating with small nail-shaped protuberance, in female unmodified; antennomeres X 1.21 longer than wide; terminal antennomeres about 1.27 times longer than wide.

Pronotum about as wide as long, wrinkly, with weak lateral swellings before lateral foveae (Fig. 10); lateral and median setose foveae well-defined; median sulcus thin.

Legs long and slender; protrochanters with large apical spine; profemora with longer spine in middle of its length; mesotrochanters at apex with two minuscule (male) or two strong and one minuscule (female) spines; mesofemora with minuscule spine at basal third.

Abdomen slightly wider than elytra; first visible abdominal tergite (IV) finely punctate, with dense, very short golden setae; carinae short, distance between carinae 0.49 of maximal tergal width. Aedeagus (Fig. 26) 0.56 mm long; median lobe weakly narrowed apically, with short and large apical lobe; endophallus with two spines; ventral spine large, forming narrow curved plate; dorsal spine slender, evenly curved, acute at apex; parameres long, overlapping apical lobe, enlarged at apex.

#### Differential diagnosis.

*Lasinus yakushimanus* is close to *Lasinus okinawanus* by sharing the presence of weak lateral swelling on the pronotum, and to *Lasinus monticola* by the presence of a nail-shaped protuberance on antennomeres IX, it differs from both by the shapes of the antennae and the aedeagus.

#### Etymology.

The name is associated with the name of the locality, Yakushima, where the speciments was found.

#### Distribution.

Japan (Yaku-shima Island).

### Lasinus amamianus sp. n.

http://zoobank.org/60DB4FAF-381E-45EC-9C69-6C3893DDE8BA

http://species-id.net/wiki/Lasinus_amamianus

Figs 17 , 27

#### Material examined.

(11 ♂♂, 9 ♀♀).HOLOTYPE, ♂, labelled as follows: (p) [Japan, Ryukyus, Kagoshima Pref., Amami-ôshima Is., Mt. Yuidake, 10.VIII.1984, S. Nomura leg.,], red label (p) HOLOTYPE *Lasinus amamianus* sp. n., Bekchiev, Hlaváč & Nomura det., 2013. (NSMT). PARATYPES: (4 ♂♂, 4 ♀♀) same data as holotype; (1 ♂, 1 ♀) Japan, Ryukyus, Kagoshima Pref., Amami-ôshima Is., Mt. Yuidake, 15.V.1983, S. Nomura leg.; (2 ♂♂, 1 ♀) Japan, Ryukyus, Kagoshima Pref., Amami-ôshima Is., Mt. Yuidake, 8.V.1987, S. Nomura leg.; (1 ♂) Japan, Kagoshima Pref., Tokunoshima Is., Mt. Inutabudake, 3.V.1988, S. Nomura leg.; (3 ♂♂, 3 ♀♀) Japan, Kagoshima Pref., Tokunoshima Is., Yonama, 4.V.1988, S. Nomura leg. (NSMT, PCPH, NMNH, PCSK).

#### Description.

Body unicoloured, reddish-brown, elytra slightly brighter, maxillary palpi yellow dark, length 2.80–3.00 mm.

Head elongate, about 1.10 times longer than wide, as long as pronotum; median sulcus shallow, reaching level of vertexal foveae. Genae with weak protuberance, covered with erected, dense golden setae.

Antennae about 1.96 mm long (Fig. 17); scapes about 3.40 times longer than pedicels; pedicels 1.40 times shorter than antennomeres III; III slightly longer than wide; IV as long as III; antennomeres V slightly longer than wide; VI about 1.20 times longer than wide; antennomeres VII 1.2 times longer than wide; VIII 1.16 times longer than wide; IX about 1.10 times longer than wide, in male with well-developed tubercles in apical ventral part, in female unmodified; antennomeres X as wide as long; terminal antennomeres about 1.47 times longer than wide.

Pronotum about as wide as long, wrinkly, with weak lateral swellings before lateral foveae; lateral and median setose foveae well-defined; median sulcus thin and deep.

Legs long and slender; protrochanters with large apical spine; profemora with long spine in middle; mesotrochanters at apex with one (male) or two (female) spines; mesofemora with minuscule spine at basal third.

Abdomen slightly wider than elytra, first visible abdominal tergite (IV) finely punctate with dense and long, golden setae; carinae short, distance between them 0.47 of maximal tergal width. Aedeagus (Fig. 27) 0.59 mm long; median lobe weakly narrowed apically, with long and narrow apical lobe; endophallus with two spines and one small lamella; ventral spine large, enlarged, forming large plate, acute at left apex; dorsal spine very big, acute at apex; lamella large, finely dentate on apical part; parameres short and slender, reaching apical lobe.

#### Differential diagnosis.

*Lasinus amamianus* and *Lasinus saoriae* differ from all other species of the genus by the shape of antennae, especially by the presence of tubercles on antennomeres IX. *Lasinus amamianus* can be readily separated from *Lasinus saoriae* by the proportion of antennomeres VII and VIII and by the shape of aedeagus.

#### Etymology.

The species name is associated with the name of the locality, Amami-ôshima Island, where most of the specimens were found.

#### Distribution.

Japan (Amami-ôshima, Tokunoshima Islands).

### Lasinus saoriae sp. n.

http://zoobank.org/D5F88EC3-FC0E-4ACE-8EEF-805AD708DFB8

http://species-id.net/wiki/Lasinus_saoriae

Figs 18 , 28

#### Type material

(6 ♂♂, 6 ♀♀). HOLOTYPE, ♂, labelled as follows: (p) [Japan, Ryukyus, Okinawa Is., Kunigami-son, Mt. Yonahadake, 22.III.2005, S. Nomura leg.], red label (p) HOLOTYPE *Lasinus saoriae* sp. n., Bekchiev, Hlaváč & Nomura det., 2013. (NSMT). PARATYPES: (2 ♂♂, 5 ♀♀) same data as holotype; (2 ♂♂, 1 ♀) Japan, Okinawa, Yona-Kunigami, 15.III.1985, leg. S. Nomura; (1 ♂) Japan, Okinawa, Kunigami, 16.III.1985, leg. S. Nomura (NSMT, PCPH, NMNH).

#### Description.

Body unicoloured, reddish-brown, maxillary palpi yellow dark, length 2.70–3.10 mm.

Head elongate, about 1.08 longer than wide, slightly shorter than pronotum; median sulcus shallow, reaching level of vertexal foveae. Genae with weak protuberance, covered with erected, dense golden setae.

Antennae about 1.8 mm long (Fig. 18); scapes long, about twice longer than pedicels; pedicels 1.22 times longer than antennomeres III; antennomeres IV as long as wide; antennomeres V slightly longer than wide; antennomeres VI about 1.33 times longer than wide; antennomeres VII and VIII slightly longer than wide; VII 0.88 times longer than wide; VIII 0.9 times longer than wide; IX about 1.26 times longer than wide, in male with short tubercles in apical ventral part, in female unmodified; antennomeres X about 1.33 longer than wide; terminal antennomeres about as long as wide.

Pronotum slightly longer than wide, wrinkly, evenly rounded before lateral foveae; lateral and median setose foveae well-defined; median sulcus thin and deep.

Legs long and slender; protrochanters with large apical spine; profemora with long spine in middle of its length; mesotrochanters at apex with one (male) or three (female) spines; mesofemora with minuscule spine at basal third.

Abdomen slightly wider than elytra; first visible abdominal tergite (IV) finely punctate, with sparse golden setae; carinae very small, distance between them 0.48 of maximal tergal width. Aedeagus (Fig. 28) 0.61 mm long; median lobe weakly narrowed apically; endophallus with two spines and two lamellas; ventral spine large, enlarged in middle, acute at apex; dorsal spine slender, acute at apex; dorsal lamella small, finely dentate on apical part; ventral lamella large; parameres very short and slender, not reaching apical lobe.

#### Differential diagnosis.

*Lasinus saoriae* strongly resembles *Lasinus amamianus* from which it differs essentially by the shapes of the antennae and the aedeagus.

#### Etymology.

The specific epithet is dedicated to Saori Takeuchi (Japan), a family friend of the first author.

#### Distribution.

Japan (Okinawajima Island).

### Lasinus okinawanus sp. n.

http://zoobank.org/56DFA417-857B-4595-8FEB-7D875CD9C8EC

http://species-id.net/wiki/Lasinus_okinawanus

Fig. 19 , 29

#### Type material.

(4 ♂♂, 4 ♀♀). HOLOTYPE, ♂, labelled as follows: (p) [Japan, Ryukyus, Okinawa Is., Okinawajima, Mt. Oppadake, Nakijin-son, 26.VI.1998, S. Nomura leg.], red label (p) HOLOTYPE *Lasinus okinawanus* sp. n., Bekchiev, Hlaváč & Nomura det., 2013 (NSMT). PARATYPES: (2 ♂♂, 3 ♀♀) same data as holotype; (1 ♂, 1 ♀) Japan, Ryukyus, Okinawa Pref., Nago-shi, Mt. Nagodake., 2.IX.2006, S. Nomura leg. (NSMT, PCPH, NMNH).

#### Description.

Body unicoloured, light reddish-brown, maxillary palpi yellow, length 3.0–3.2 mm.

Head elongate, about 1.03 longer than wide, and as long as pronotum; median sulcus visible on rostrum, on vertex reaching level of vertexal foveae. Genae with weak protuberance, covered with erected, dense golden setae.

Antennae long about 1.94 mm (Fig. 19); scapes 1.16 times longer than wide, 1.40 times as long as pedicels; pedicels as long as wide and as long as antennomeres III; antennomeres IV 1.25 times shorter than III; antennomeres V 1.20 times longer than wide; antennomeres VI 1.60 times longer than wide; antennomeres VII as long as wide; antennomeres VIII about 1.40 times longer and distinctly wider than VII; antennomeres IX as long as wide and about same length as terminal antennomeres, in male with large, shallow, in apical half highly inclined discoidal plate, in female unmodified; antennomeres X quadrate; terminal antennomeres 1.60 times as long as X and about 1.37 times longer than wide.

Pronotum about as wide as long, wrinkly, with weak lateral swellings before lateral foveae; lateral and median setose foveae well-defined; median longitudinal sulcus present.

Legs long and slender; protrochanters with large apical spine; profemora with longer spine in middle of its length; mesotrochanters at apex with one (male) or three (female) spines; mesofemora with minuscule spine at basal third.

Abdomen slightly wider than elytra, first visible abdominal tergite (IV) finely punctate with sparse, short golden setae; carinae short, distance between them 0.39 of maximal tergal width. Aedeagus (Fig. 29) 0.60 mm long; median lobe weakly narrowed apically, with long and narrow apical lobe; endophallus with two spines and two lamellas; ventral spine very large, short, curved downwards in middle, acute rightward at apex; dorsal spine very strong, acute at apex; dorsal lamella small finely dentate on apical part; ventral lamella large; parameres long and slender, different in length, left one overlapping apical lobe, right one shorter,.

#### Differential diagnosis.

*Lasinus okinawanus* is most closely related to *Lasinus spinosus* by the antennomeres IX with a shallow cavity, and the lack of an apical nail-shaped protuberance. It differs from latter by antennomeres IX having highly inclined shallow discoidal plate in apical half, and by the shape of the aedeagus.

#### Etymology.

The specific name is associated with the name of the type locality, Okinawa Island, where the type specimens were found.

#### Distribution.

Japan (Okinawajima Island).

## Supplementary Material

XML Treatment for Lasinus

XML Treatment for Lasinus mandarinus

XML Treatment for Lasinus spinosus

XML Treatment for Lasinus monticola

XML Treatment for Lasinus sinicus

XML Treatment for Lasinus mikado

XML Treatment for Lasinus yamamotoi

XML Treatment for Lasinus inexpectatus

XML Treatment for Lasinus yakushimanus

XML Treatment for Lasinus amamianus

XML Treatment for Lasinus saoriae

XML Treatment for Lasinus okinawanus
